# Supplementary material for: Graphene Oxide Carboxymethylcellulose Nanocomposite for Dressing Materials
Source: Materials (Basel). 2020 Apr 23;13(8):1980. doi: 10.3390/ma13081980 (PMC7216044; doi:10.3390/ma13081980)
Supplement: Supplementary file 1 [file materials-13-01980-s001.pdf]

materials

Supplementary Information

Graphene Oxide Carboxymethylcellulose Nanocomposite for Dressing Materials

Maria Luisa Saladino [1,*], Marta Markowska [2,3], Clara Carmone [1], Patrizia Cancemi [1], Rosa Alduina [1], Alessandro Presentato [1], Roberto Scaffaro [4], Dariusz Biały [3,5,*], Mariusz Hasiak [6], Dariusz Hreniak [2,3] and Magdalena Wawrzyńska [3,5,*]

1 Department of Biological, Chemical and Pharmaceutical Sciences and Technologies (STEBICEF), University of Palermo, Viale delle Scienze Bld. 16-17, I-90128 Palermo, Italy; claracarmone@gmail.com (C.C.); patrizia.cancemi@unipa.it (P.C.); valeria.alduina@unipa.it (R.A.); alessandro.presentato@unipa.it (A.P.)
2 Institute of Low Temperature and Structure Research, Polish Academy of Sciences, Okólna 2, PL-50-422 Wrocław, Poland; m.markowska@intibs.pl (M.M.); d.hreniak@intibs.pl (D.H.)
3 Carbonmed Spółka z ograniczoną odpowiedzialnością, ul. Okólna 2, 50-422 Wrocław, Poland
4 Department of Engineering, University of Palermo, Viale delle Scienze Bld. 6, I-90128 Palermo, Italy; roberto.scaffaro@unipa.it
5 Division of Preclinical Research, Faculty of Health Sciences, Wroclaw Medical University, Ludwika Pasteura 1, PL-50-367 Wrocław, Poland
6 Department of Mechanics and Material Science Engineering, Wrocław University of Science and Technology, Smoluchowskiego 25, PL-50-370 Wrocław, Poland; mariusz.hasiak@pwr.edu.pl
* Correspondence: marialuisa.saladino@unipa.it (M.L.S.); dariusz.bialy@umed.wroc.pl (D.B.); magdalena.wawrzynska@umed.wroc.pl (M.W.)

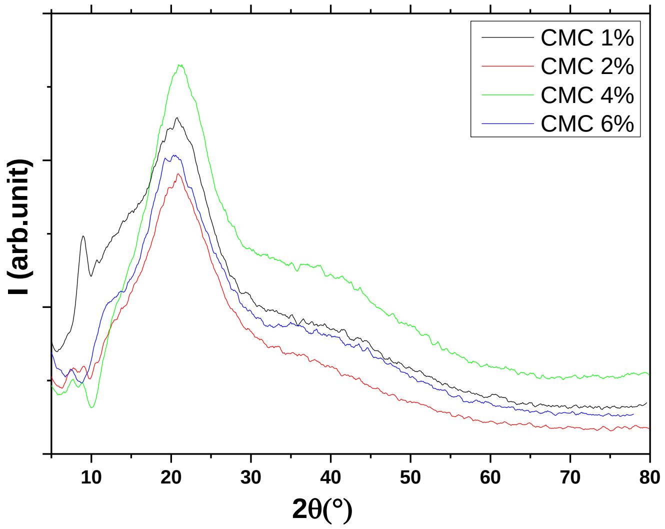

Figure S1. XRD patterns of cellulose films.

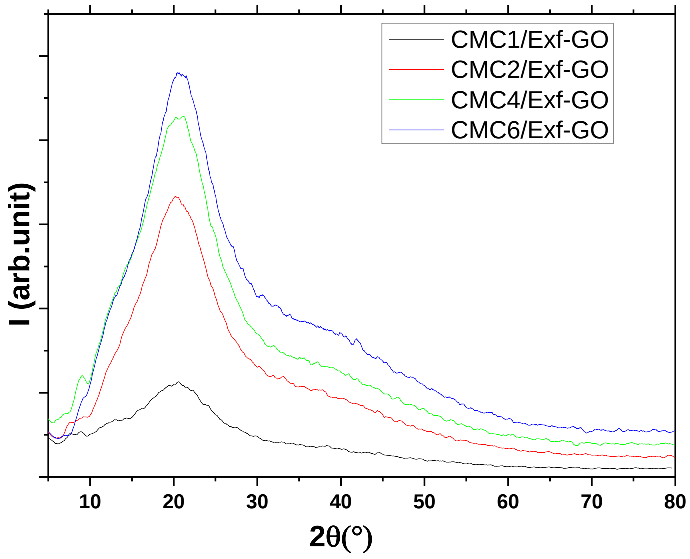

Figure S2. XRD patterns of CMCx/*Exf*-GO nanocomposites.

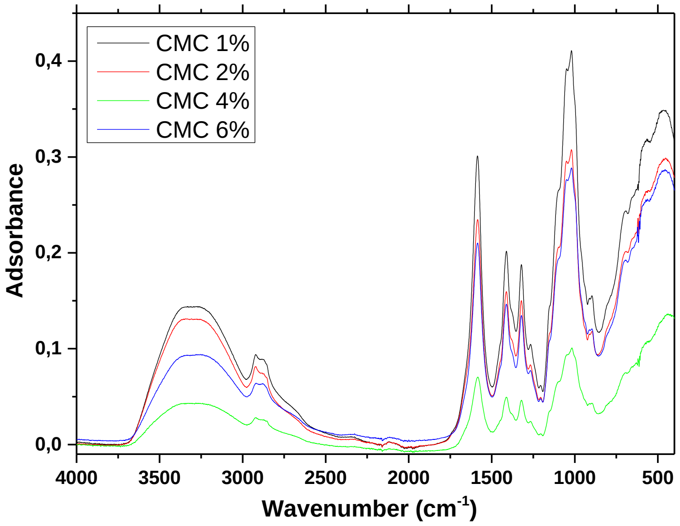

Figure S3. IR spectra of cellulose films.

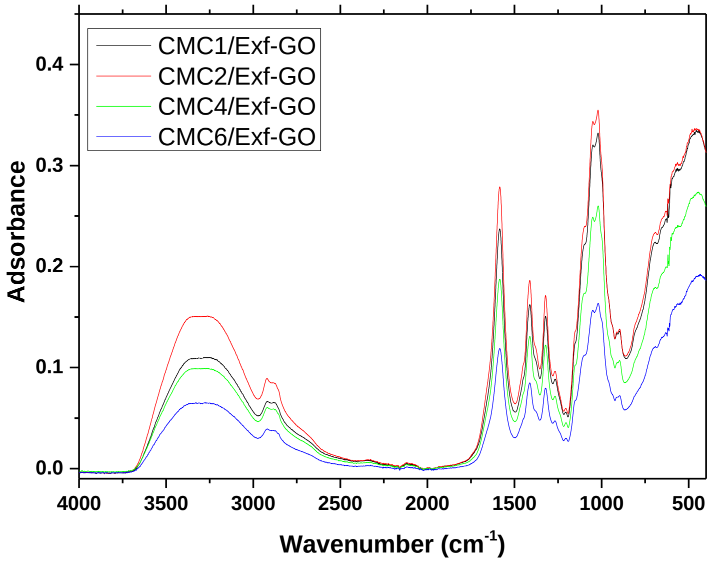

Figure S4. IR spectra of CMCx/*Exf*-GO nancocomposites.

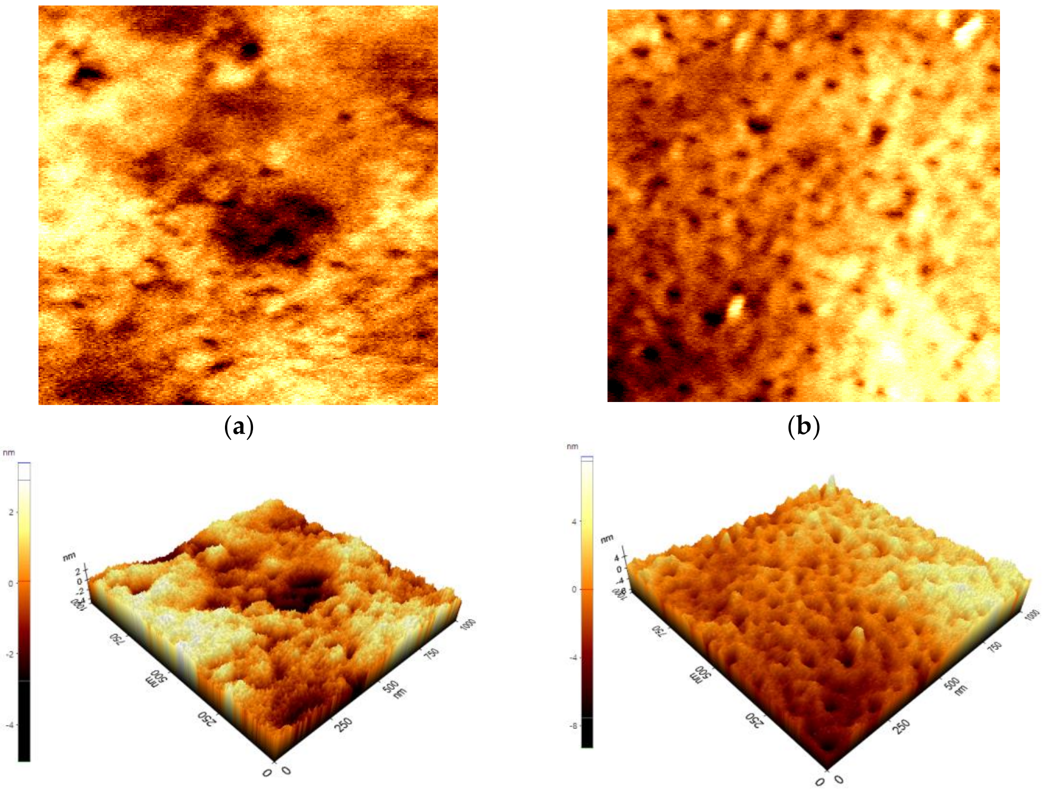

(**a**) (**b**)

(**c**) (**d**)

Figure S5. 2D (**a**, **b**) and 3D (**c**, **d**) AFM topography images for the CMC 1% film (**a**, **c**) and CMC1/*Exf*-GO nanocomposite (**b**, **d**)) recorded in contact mode for scanning area of 1 µm × 1 µm.

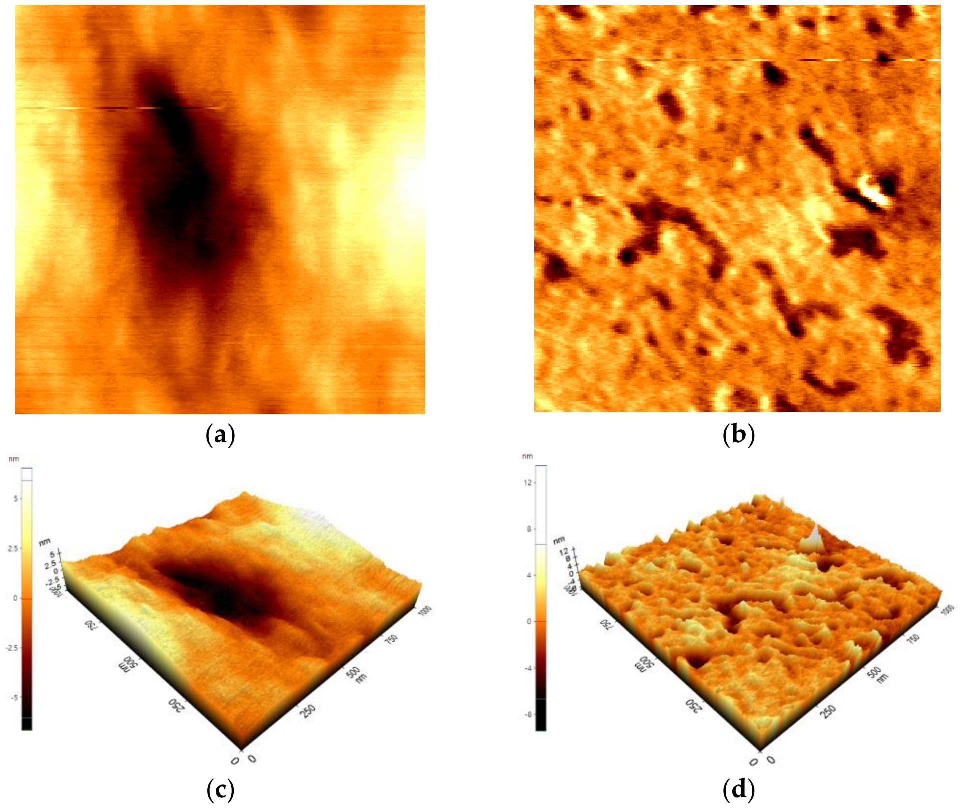

Figure S6. 2D (**a**, **b**) and 3D (**c**, **d**) AFM topography images for the CMC 2% films (**a**, **c**) and CMC2/Exf-GO nanocomposites (**b**, **d**) recorded in contact mode for scanning area of 1 µm × 1 µm.

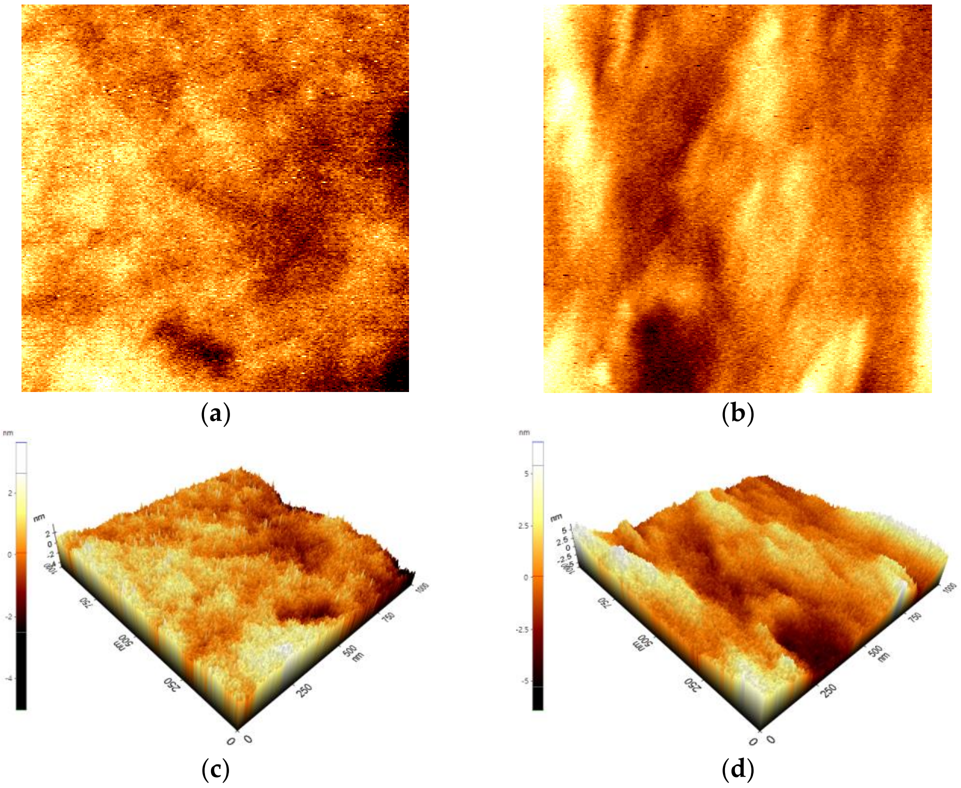

Figure S7. 2D (**a**, **b**) and 3D (**c**, **d**) AFM topography images for the CMC 4% films (**a**, **c**) and CMC4/Exf-GO nanocomposites (**b**, **d**) recorded in contact mode for scanning area of 1 µm × 1 µm.

© 2020 by the authors. Submitted for possible open access publication under the terms and conditions of the Creative Commons Attribution (CC BY) license (http://creativecommons.org/licenses/by/4.0/).
